# Supplementary material for: Phosphite Reduces the Predation Impact of Poterioochromonas malhamensis on Cyanobacterial Culture
Source: Plants (Basel). 2021 Jul 2;10(7):1361. doi: 10.3390/plants10071361 (PMC8309446; doi:10.3390/plants10071361)
Supplement: Supplementary file 1 [file plants-10-01361-s001.zip › plants-1276897-supplementary.pdf]

Supplementary Information for

Phosphite reduces the predation impact of *Poterioochromonas malhamensis* on cyanobacterial culture

Narumi Toda, Hiroki Murakami, Akihiro Kanbara, Akio Kuroda, Ryuichi Hirota*

Unit of Biotechnology, Division of Biological and Life Sciences, Graduate School of Integrated Sciences for Life, Hiroshima University, Hiroshima, Japan

*** Correspondence :**

Ryuichi Hirota

hirota@hiroshima-u.ac.jp

Table S1. Sampling locations and their information of the environmental water samples.

Location	Water sample	Location name	GPS position	Date	Clearance time	Ochromonadale detection [a]
1	1	Budouike Pond st1	34.403059,132.716420	2018.05.11	2.2	N.D.
	2	Budouike Pond st2	34.402337,132.715227	2018.05.11	2.4	+
	3	Budouike Pond st3	34.402682,132.715506	2018.05.11	8.3	+
	4	Budouike Pond st4	34.401049,132.713009	2018.05.11	2.0	+
	5	Budouike Pond st5	34.400190,132.712397	2018.11.27	2.7	N.E.
	6	Budouike Pond st5	34.400190,132.712397	2019.01.28	1.6	N.E.
	7	Budouike Pond st6	34.398809,132.712258	2017.12.08	N.E.	+
	8	Budouike Pond st6	34.398809,132.712258	2018.05.11	1.9	-
	9	Budouike Pond st6	34.398809,132.712258	2018.06.26	4.6	-
	10	Budouike Pond st6	34.398809,132.712258	2019.01.19	3.0	N.E.
2	11	Kurosegawa River (PointC)	34.387663,132.732964	2017.12.08	N.E.	+
	12	Kurosegawa River (PointC)	34.387663,132.732964	2018.01.30	3.0	N.E.
	13	Kurosegawa River (PointC)	34.387663,132.732964	2018.06.26	2.0	N.D.
	14	Kurosegawa River (PointC)	34.387663,132.732964	2018.08.10	1.0	N.E.
	15	Kurosegawa River (PointC)	34.387663,132.732964	2018.10.18	1.9	N.E.
	16	Kurosegawa River (PointC)	34.387663,132.732964	2019.01.18	1.9	N.E.
3	17	Okudaoike Pond	34.387663,132.732964	2017.12.08	N.E.	+
4	18	Azumako Waterfall	34.396578,132.739080	2018.11.27	2.6	N.E.
	19	Azumako Waterfall	34.396578,132.739080	2019.01.18	1.7	N.E.
5	20	Sennennike Pond	34.385459,132.748886	2018.06.26	2.3	-
6	21	Minaga River	34.395826,132.767844	2018.11.27	2.5	N.E.
7	22	Shitami Pond	34.407865,132.705917	2018.11.27	2.6	N.E.
8	23	Kineharagawa River	34.447330,132.789419	2018.12.20	1.3	N.E.
9	24	Science Park Pond	34.399177,132.733570	2020.08.30	N.E.	+
10	25	Artificial biotope	34.433251,132.747511	2020.08.25	N.E.	+

[a] N.E. and N.D. stand for Not Examined and Not Detected, respectively. The symbol + and – stand for the detection of Ochromonadales and other protists, respectively.

Table S2. Time lapse video recording data and observed clearance time of *Syn* 7942 culture using environmental water samples.

Serial number of samples	Sampling location		GPS position	P conc. (mM)	Pi				Pt			
					Prey cells	Start date	End date	Clearance time (d)[a]	Prey cells	Start date	End date	Clearance time (d)[a]
1	1	Budouike Pond	34.403059, 132.716420	0.2	*Syn* 7942	2018/10/18 18:47	2018/10/21 13:57	2.80	AK024	2018/10/18 18:47	2018/10/21 3:27	2.36
				2		2018/10/18 18:47	2018/10/21 3:57	2.38		2018/10/18 18:47	2018/10/22 19:57	4.05
				10		2018/10/18 18:47	2018/10/22 00:00	3.22		2018/10/18 18:47	2018/10/21 20:07	3.06
				20		2018/10/18 18:47	2018/10/22 4:57	3.42		2018/10/18 18:47	2018/10/23 16:37	4.91
2	2	Sennnenike Pond	34.385459, 132.748886	0.2	*Syn* 7942	2018/11/28 17:08	2018/11/29 13:38	0.85	AK024	2018/11/28 17:08	2018/12/2 5:38	3.52
				2		2018/11/28 17:08	2018/12/5 18:28	7.06		2018/11/28 17:08	2018/12/1 14:38	2.90
				10		2018/11/28 17:08	2018/12/3 2:18	4.38		2018/11/28 17:08	2018/12/2 5:38	3.52
				20		2018/11/28 17:08	2018/12/6 14:58	7.91		2018/11/28 17:08	2018/12/6 14:58	7.91
3	3	Science Park Pond	34.399177, 132.733570	0.2	*Syn* 7942	2018/12/21 16:20	2018/12/24 12:40	2.85	AK024	2018/12/21 16:20	2018/12/23 20:01	2.15
				2		2018/12/21 16:20	2018/12/24 6:00	2.57		2018/12/21 16:20	2018/12/24 1:01	2.36
				10		2018/12/21 16:20	2018/12/23 18:20	2.08		2018/12/21 16:20	2018/12/25 8:41	3.68
				20		2018/12/21 16:20	2018/12/25 13:40	3.89		2018/12/21 16:20	2018/12/25 3:41	3.47
4	4	Kinehara River	34.447330, 132.789419	0.2	*Syn* 7942	2018/12/21 16:19	2018/12/22 22:19	1.25	AK024	2018/12/21 16:19	2018/12/21 16:19	0.00
				2		2018/12/21 16:19	2018/12/22 22:19	1.25		2018/12/21 16:19	2018/12/23 19:59	2.15
				10		2018/12/21 16:19	2018/12/23 6:39	1.60		2018/12/21 16:19	2018/12/24 10:59	2.78
				20		2018/12/21 16:19	2018/12/23 18:19	2.08		2018/12/21 16:19	2018/12/25 1:59	3.40
5	5	Kurosegawa River (Point A)	34.439530, 132.721366	0.2	*Syn* 7942	2018/10/18 18:46	2018/10/21 04:46	2.42	AK024	2018/10/18 18:46	2018/10/20 21:46	2.13
				2		2018/10/18 18:46	2018/10/21 8:56	2.59		2018/10/18 18:46	2018/10/21 17:06	2.93
				10		2018/10/18 18:46	2018/10/21 17:06	2.93		2018/10/18 18:46	2018/10/22 11:06	3.68
				20		2018/10/18 18:46	2018/10/23 10:36	4.66		2018/10/18 18:46	2018/10/22 5:36	3.45
6				0.2	*Syn* 7942	2018/10/18 18:47	2018/10/21 03:56	2.38	AK024	2018/10/18 18:47	2018/10/20 11:46	1.71
				2		2018/10/18 18:47	2018/10/21 8:26	2.57		2018/10/18 18:47	2018/10/21 8:26	2.57
				10		2018/10/18 18:47	2018/10/21 16:16	2.90		2018/10/18 18:47	2018/10/22 1:36	3.28
				20		2018/10/18 18:47	2018/10/23 8:16	4.56		2018/10/18 18:47	2018/10/21 23:56	3.21
7				0.2	*Syn* 7942	2018/11/28 17:08	2018/11/30 21:27	2.18	AK024	2018/11/28 17:08	2018/11/30 15:38	1.94
				2		2018/11/28 17:08	2018/11/30 14:27	1.89		2018/11/28 17:08	2018/11/30 18:48	2.07
				10		2018/11/28 17:08	2018/11/30 17:57	2.03		2018/11/28 17:08	2018/12/1 9:48	2.69
				20		2018/11/28 17:08	2018/12/1 1:27	2.35		2018/11/28 17:08	2018/12/2 2:58	3.41
8	6	Kurosegawa River (Point B)	34.422788, 132.740828	0.2	*Syn* 7942	2018/8/10 17:35	2018/8/12 2:55	1.39	AK024	2018/8/10 17:35	2018/8/10 17:35	0.00
				2		2018/8/10 17:35	2018/8/12 6:15	1.53		2018/8/10 17:35	2018/8/12 6:15	1.53
				10		2018/8/10 17:35	2018/8/12 17:15	1.99		2018/8/10 17:35	2018/8/13 20:05	3.10
				20		2018/8/10 17:35	2018/8/13 23:25	3.24		**2018/8/10 17:35**	**2018/8/24 18:45**	**14.00**
9				0.2	*Syn* 7942	2018/8/10 17:35	2018/8/12 11:45	1.76	AK024	2018/8/10 17:35	2018/8/12 2:55	1.39
				2		2018/8/10 17:35	2018/8/12 11:45	1.76		2018/8/10 17:35	2018/8/14 18:15	4.03
				10		2018/8/10 17:35	2018/8/13 21:05	3.15		2018/8/10 17:35	2018/8/13 22:15	3.19
				20		2018/8/10 17:35	2018/8/14 4:55	3.47		**2018/8/10 17:35**	**2018/8/24 18:45**	**14.00**

Table S2. *continued*

10	7	Kurosegawa River (Point C)	34.387663, 132.732964	0.2	*Syn* 7942	2021/1/30 15:49	2021/2/2 18:50	3.13	RH714	2021/1/30 15:49	2021/2/2 2:09	2.43
				2		2021/1/30 15:49	2021/2/2 10:50	2.79		2021/1/30 15:49	2021/2/2 3:29	2.49
				10		2021/1/30 15:49	2021/2/2 17:10	3.06		2021/1/30 15:49	2021/2/2 9:19	2.73
				20		2021/1/30 15:49	2021/2/3 21:10	4.22		2021/1/30 15:49	2021/2/12 5:29	12.57
11				0.2	*Syn* 7942	2021/3/17 10:17	2021/3/19 15:57	2.24	RH714	2021/3/17 10:17	2021/3/18 7:04	0.87
				2		2021/3/17 10:17	2021/3/20 4:17	2.75		2021/3/17 10:17	2021/3/20 8:14	2.91
				10		2021/3/17 10:17	2021/3/20 9:17	2.96		2021/3/17 10:17	2021/3/21 5:24	3.80
				20		2021/3/17 10:17	2021/3/21 16:57	4.28		**2021/3/17 10:17**	**2021/4/6 8:54**	**14.00**

[a] Bold faces indicate the completion of *Syn* 7942 culture without the clearance by grazers.
